# NF-kappaB: Two Sides of the Same Coin

**DOI:** 10.3390/genes9010024

**Published:** 2018-01-09

**Authors:** Bruno R. B. Pires, Rafael C. M. C. Silva, Gerson M. Ferreira, Eliana Abdelhay

**Affiliations:** 1 Laboratório de Célula-Tronco, Instituto Nacional de Câncer, 20230-130 Rio de Janeiro, RJ, Brazil; gerson.ferreira@inca.gov.br (G.M.F.); eabdelhay@inca.gov.br (E.A.); 2 CAPES Foundation, Ministério da Educação, 70040-020 Brasília, DF, Brazil; rcmcs27@hotmail.com

**Keywords:** NF-kB, NF-kappaB, signaling, cancer, inflammation, immune response, treatment, therapy

## Abstract

Nuclear Factor-kappa B (NF-κB) is a transcription factor family that regulates a large number of genes that are involved in important physiological processes, including survival, inflammation, and immune responses. More recently, constitutive expression of NF-κB has been associated with several types of cancer. In addition, microorganisms, such as viruses and bacteria, cooperate in the activation of NF-κB in tumors, confirming the multifactorial role of this transcription factor as a cancer driver. Recent reports have shown that the NF-κB signaling pathway should receive attention for the development of therapies. In addition to the direct effects of NF-κB in cancer cells, it might also impact immune cells that can both promote or prevent tumor development. Currently, with the rise of cancer immunotherapy, the link among immune cells, inflammation, and cancer is a major focus, and NF-κB could be an important regulator for the success of these therapies. This review discusses the contrasting roles of NF-κB as a regulator of pro- and antitumor processes and its potential as a therapeutic target.

## 1. Evolutionary Origin of Nuclear Factor-kappa B Family and Its Signaling Pathway

Dorsal is a transcription factor that was originally discovered to be responsible for dorsoventral polarity during the early stages of *Drosophila* sp. development. Its activation occurs in the ventral region of the egg when the receptor toll binds to the ligand spätzle, which triggers signal transduction that culminates in the destruction of cactus, which is an inhibitory protein that binds to dorsal in the cytoplasm. Therefore, the released dorsal is transported to the nucleus and exerts DNA-binding activity [1,2,3]. The cactus-dorsal system is evolutionarily conserved from fly to vertebrates, and is also crucial for the immune response. The vertebrate ortholog of dorsal is the Nuclear Factor-kappa B (NF-κB) family; and cactus corresponds to inhibitor of NF-κB (IκB) [4]. 

Five genes encode the family of NF-κB transcription factors: *NFKB1*, *NFKB2*, *RELA*, *RELB,* and *REL*, the protein products of which are p50, p52, p65 (RelA), RelB, and c-Rel, respectively. *NFKB1* and *NFKB2* are expressed as the precursors p105 and p100, which are cleaved to the functional transcription factors p50 and p52, respectively [5]. The NF-κB members are ubiquitously expressed, but their functionality might depend on specific cellular stimuli [6]. These transcription factors generally act as homo- or heterodimers, and the combination of every member is possible, although some of them have greater affinity. For instance, due to their structure, p65/p50 and RelB/p52 are the more stable dimers [7,8]. 

The NF-κB family and dorsal share an evolutionarily conserved N-terminal region, known as the Rel homology domain (RHD), which is essential for homo- and heterodimerization, nuclear targeting, and binding to DNA or IκB (Cactus, in *Drosophila* sp.). The C-terminal region, where the transcriptional activation domain (TAD) is located, is not conserved among the NF-κB members because p50 and p52 are products of partial proteolysis, and thus have a glycine-rich region instead of TAD [9,10].

Activation of the NF-κB pathway can be initiated by a large number of extracellular stimuli, but they have a similar signal transduction cascade resulting basically from phosphate transference. This signaling comprises canonical (classical) and non-canonical (alternative) pathways.

**Figure 1:**
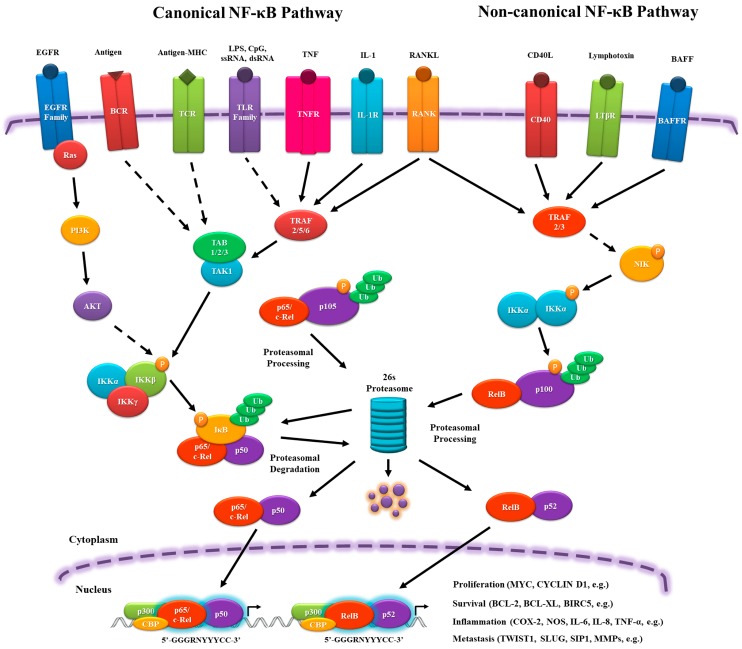
Schematic overview of canonical and non-canonical Nuclear Factor-kappa B (NF-κB) pathways. EGF, epidermal growth factor; BCR, B-cell receptor; TCR, T-cell receptor; MHC, major histocompatibility complex; LPS, lipopolysaccharide; ssRNA, single-stranded RNA; dsRNA, double-stranded RNA; TNF, tumor necrosis factor; IL-1, interleukin-1; RANKL, receptor activator of nuclear factor κ-B ligand; CD40, cluster of differentiation 40; LTβR, lymphotoxin beta receptor; BAFF, B-cell activating factor; PI3K, phosphatidylinositide 3-kinase; TRAF, TNF receptor associated factor; NIK, NF-κB-inducing kinase; IκB, inhibitor of NF-κB; IKK, IκB kinase; CBP, CREB-binding protein; BCL-2, B-cell lymphoma 2; BCL-XL, B-cell lymphoma-extra large; COX-2, ciclo-oxigenase-2; NOS, nitric oxide synthase; SIP1, smad interacting protein 1 (ZEB2); MMP, Matrix metalloproteinase; P, phosphate group; Ub, ubiquitin moieties.

In the canonical pathway, NF-κB dimers are regulated by inhibitory molecules of the IκB family, which prevent their translocation into the nucleus, interacting, and forming a stable complex similarly to cactus-dorsal. To release the NF-κB complex, signaling pathways are activated by pro-inflammatory cytokine receptors, such as tumor necrosis factor receptor (TNFR), IL-1 receptor (IL-1R), and toll-like receptor (TLR) family members (TLR3, TLR4, TLR7); antigen receptors, such as T cell receptor (TCR) and B cell receptor (BCR); and, growth factors, such as Epidermal growth factor receptor (EGFR) family members. These receptors are able to activate the IκB kinase (IKK) complex (IKKα, IKKβ and IKKγ (NF-κB essential modulator (NEMO))), which phosphorylates and facilitates the ubiquitination of IκB and its subsequent degradation by the 26s proteasome. The dimers p65/p50 and c-Rel/p50 are then translocated into the nucleus and activate target gene expression [9,11,12,13] (Figure 1).

In the non-canonical pathway, there is no IκB, but the RelB/p100 complex is inactive in the cytoplasm. Signaling from cluster of differentiation 40 ligand (CD40L), lymphotoxin β receptor (LTβR), and B cell–activating factor receptor (BAFFR) leads to NF-κB-inducing kinase (NIK) activation, phosphorylating the homodimer IKKα/IKKα, which transfers the phosphate group to the C-terminal residues of p100 to be ubiquitinated and proteasomally processed into p52. It ultimately leads to the nuclear translocation of RelB/p52 and the induction of target gene expression [9,11,12,14] (Figure 1).

In the nucleus, the NF-κB complex binds to sequence-specific target DNA, known as κB sites, (5′-GGGRNYYYCC-3′, where R: purine, Y: pyrimidine and N: any nucleotide), which are present in promoters of the target genes, assemble with the basal transcriptional machinery, and might associate with other transcription factors, including AP-1 (c-Jun/c-Fos complex) and chromatin remodeling proteins, such as CREB-binding protein (CBP) and p300 (Figure 1). In addition, NF-κB can be influenced by other transcription factors that can interact physically, altering its ability to bind to the DNA and genes that will be activated [5,15,16,17,18,19,20,21,22]. This variety of players that can influence and activate NF-κB nuclear translocation will be responsible for the diverse and sometimes opposing roles of NF-κB as a pro- and an anti-inflammatory mediator.

## 2. The Role of NF-κB in Physiology, Inflammation and Cancer

NF-κB is recognized as a crucial component of many immune responses. Innate immune cells, such as macrophages and dendritic cells (DCs), rely on NF-κB for the secretion of pro-inflammatory cytokines after pattern recognition receptor (PRR) activation [23]. NF-κB is also recognized as an important anti-apoptotic transcription factor for immune cells, such as neutrophils, and it is essential for the development of lymphocytes [24,25,26,27,28,29]. Furthermore, the adaptive immune response activation and polarization also seem to be dependent on NF-κB [26,30]. Professional antigen presenting cells (usually activated DCs) will make the link between innate and adaptive immunity, presenting antigens and activating clonal receptors (which suffered gene segment recombination during T cell development) and its co-receptors from specific T lymphocytes (CD4 for T CD4+ cells and CD8 for T CD8+ lymphocytes), and also secreting cytokines that will drive the activation of TCD4+ cells on T helpers (Th) from the major sub-types, Th1, Th2, Th9, Th17, Th22, T follicular helpers (Tfh), and T regulatory (Treg) cells [31,32]. These sub-types are responsible for orchestrating the adaptive immune response, secreting cytokines, and activating and stimulating proper immune cells, including B cells (responsible for antibody secretion), which will afford protection against the specific insulting agent. Each sub-type of T CD4+ affords protection, in general, to different types of infection [33]. NF-κB has been described as a crucial component in many of these sub-types of polarization (Th1, Th2, Th17, Th9) [34,35,36,37,38,39]. Box 1 summarizes the diverse effects of NF-κB on the immune system.

Box 1 **Main effects of Nuclear Factor-kappa B (NF-κB) on the immune system.**

***Adaptive immunity***

Development: NF-κB is crucial to B and T cell lymphocytes development, promoting transcription of survival and maturation factors [25,40]. It also participates in secondary lymphoid organogenesis after cytokine receptors activation [41,42].

Functions: Supports the activity of different T CD4+ cell sub-types, such as T helper 1 (Th1) and T regulatory cells (Tregs) [43]. The p65 (RelA) subunit is crucial for survival of activated TCD8+ cells [44]. It is important to costimulatory receptors functions, such as CD28 [45].

***Innate Immunity***

Development: NF-κB is important to myeloid progenitor differentiation and Granulocyte-macrophage colony-stimulating factor (GMCSF) mediated signaling. The development of different cells derived from myeloid progenitors are specifically influenced by different members of the NF-κB family of transcription factors [46,47].

Proinflammatory functions: NF-κB is crucial for certain cytokines secretion after pattern recognition receptor (PRR) activation [48]. It is a fundamental part of cytokine (and chemokine) responses, and IgE mediated activation of different myeloid cells, like eosinophils and mast cells [49,50]. It is also involved in perforin and IFN-γ production by natural killer (NK) cells, although one of NF-κB subunits (p50) is associated to inhibition of IFN-γ secretion and proliferation of NK cells [51], and collaborates with RIPK1 in dying cells, promoting T cell cross-presentation by dendritic cells (DCs) [52]. In addition, NF-κB cooperates with STAT1 to the development of type I DCs after IL-12 exposure [53].

Anti-inflammatory functions: Homodimers of p50, which do not possess a transactivator domain, have been described to be related to cytokine inhibition in macrophages due to nonproductive binding on different promoters [54]. Myeloid derived suppressor cells rely on NF-κB for STAT3 activation and IDO secretion [55]. IκB kinase β (IKKβ) can counteract STAT1 activation in macrophages, inhibiting its inflammatory functions [56]. At last, NF-κB is involved on neutrophils apoptosis, restraining the survival of this inflammatory cells [57].

**Figure 2:**
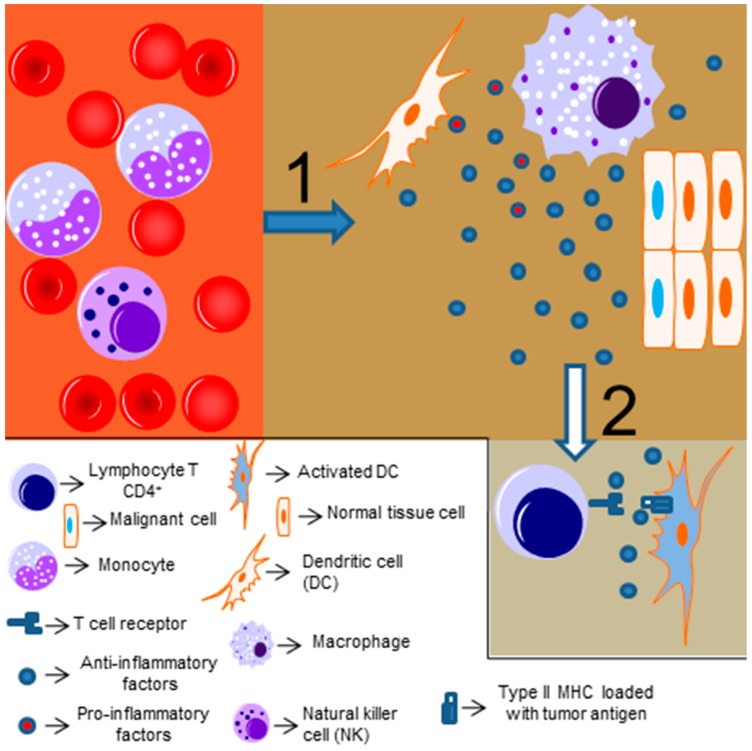
Immune response to tumors: (**1**) The loss of homeostasis driven by transformed cells will usually lead to tissue macrophage activation and an initial inflammatory response that will recruit other innate immune cells from nearby capillaries, like monocytes and Natural killer (NK) cells. After this initial phase, if the malignant cells are not cleared out, anti-inflammatory and regulatory factors, like IL-10 and TGF-β, can be secreted in order to restrain inflammation. Tumor cells can also secrete anti-inflammatory factors, rendering macrophages a tumor supportive phenotype, known as tumor associated macrophages (TAMs). (**2**) These anti-inflammatory factors will drive antigen presenting cells to promote a regulatory phenotype on naive T CD4+ lymphocytes (present in lymph nodes), which will restrain adaptive immunity to tumor antigens. The aim of immunotherapies nowadays is to revert this tumor permissive cycle, mainly through inhibition of receptors expressed by T cells that act as antitumor immunity silencers. In vitro co-cultures of macrophages and tumor cells not always support the role of tumor cells promoting an anti-inflammatory phenotype of macrophages. It seems that different tumors can induce different phenotypes on macrophages, even promoting its antitumor activity, through NF-κB. How these tumors escape from immune response is not well understood, but different approaches to unleash the tumor immunity are needed in this case, and also when there is no immune cell infiltration on the affected tissue.

The link between inflammation and cancer was realized over 150 years ago, when Virchow described that the presence of immune cells could be related to the place where the cancer would appear in an inflamed tissue [58,59,60,61,62]. Chronic inflammation (driven by infectious or non-infections insults) is associated with cancer development [63]. The exact mechanism linking inflammation and tumorigenesis is not fully understood, but it is known that reactive oxygen species and other potentially harmful mediators of the immune response might induce genetic instability and promote mutations in tissues cells [64,65]. Moreover, chronic and persistent low-level degrees of inflammation might be accompanied by infiltrating anti-inflammatory and regulatory immune cells, which will attempt to resolve the inflammatory response and induce cell proliferation and angiogenesis (as a tissue repair mechanism), thus protecting the tumor from other immune cells and providing a signal to maintain its proliferative and invasive profile [64]. This idea was reinforced by Dvorak [66], who stated that “cancers are wounds that do not heal” (Figure 2 represents a simplified scheme of the tumor immune response). Thus, depending on the level and type of inflammatory response, it can present an antitumor effect or even stimulate and promote tumor growth and invasion.

NF-κB is considered as the master regulator of the inflammatory response, and is also associated with cancer development and pathogenesis. Some of the most incident cancers, including breast, gastric, leukemia, and lymphoma, have been described to have NF-κB as a key regulator of their development and progression (as discussed below). The malignant potential of NF-κB was originally described by Beug et al. [67], who reported that the v-rel oncogene (homolog of c-Rel), according to Wilhelmsen et al. [68] of reticuloendotheliosis virus strain T was responsible for the development of aggressive lymphomas in chicken models. However, only after cancer-related inflammation (CRI) was recognized as a hallmark of cancer did NF-κB acquire new importance in cancer studies [69,70]. Numerous data have indicated a positive feedback between NF-κB activation and inflammatory signaling that favors tumor development, such as the up-regulation of cyclooxigenase (COX)-2; nitric oxide synthase (NOS); inflammatory cytokines, such as IL-6, IL-8, and TNF-α; and, chemokines, such as CCL2 and CXCL8 [9,69]. Several anti-apoptotic genes, such as *BCL-2*, *BCL-XL*, and *BIRC5* (Survivin), are regulated positively by NF-κB [9,71]. Furthermore, NF-κB also induces the expression of mitogenic proteins, such as c-Myc and Cyclin D1 [9,72]. Moreover, persistent NF-κB activation has also been related to chemo- and radiotherapy resistance through apoptosis inhibition and growth stimuli [71,73]. Furthermore, we discuss the specific role of NF-κB in different types of neoplasia.

## 3. Role of NF-κB in Hematological Malignancies

A remarkable characteristic of lymphomas and leukemias is the constitutive expression of NF-κB. NF-κB is overexpressed in primary acute myeloid leukemia cells, as well as leukemic stem cells, but not in normal hematopoietic cells [74]. In childhood acute lymphoblastic leukemia, it is constitutively expressed in the majority of cases, independently of the subtype [75]. Human T cell leukemia virus type I (HTLV-I) is the causative agent of adult T cell leukemia (ATL). HTLV-I expresses Tax protein, which is a strong oncogene that promotes the expression of viral proteins in the nucleus and controls cellular genes through the CREB/ATF-, SRF-, AP1, and NF-κB pathways. In addition, Tax triggers several cellular alterations, such as an uncontrolled cycle cell, inhibition of DNA repair, and apoptosis, leading to cancer [76]. Tax activates the canonical and non-canonical NF-κB pathway by IKK complex activation, and therefore is essential for the immortalization and survival of HTLV-I-infected T cells [77]. The activation of IKKs by Tax depends on TGF-β-activating kinase 1 (Tak1) [78], and the polyubiquitination of Tax also contributes to the activation of the IKK complex. Furthermore, Tax also interacts with TAX1BP1, inhibiting the activity of the NF-κB inhibitor A20 (also known as TNFAIP3) [79].

Constitutive expression of NF-κB inhibits apoptosis in Hodgkin/Reed-Sternberg (HRS) cell lines, stimulating cell proliferation. These cells show constitutive expression of p65 and p50 subunits [80], caused by IκBs mutations and abnormal activation of IKK proteins that lead to permanent nuclear NF-κB activity [81]. The studied cell lines showed mutations that impaired the production of a full-length IκB protein. The Hodgkin cell line L428 produces a C-terminal truncated product of 30 kDa caused by the deletion of 19 nucleotides in the intron joining exons 5 and 6. Another cell line, KMH-2, expresses an 18-kDa truncated IκB caused by deletion of 214 nucleotides of exon 3, which was replaced with a pentanucleotide, an alternative donor splice site between exon 3 and 4 and a stop codon in exon 4 [81,82]. A dominant-negative form of IκB blocked the constitutive expression of NF-κB, causing a reduction of apoptosis and repression of the proliferation and tumor growth of HRS cells in mice, confirming the role of this transcription factor as an inducer of tumorigenesis [83]. 

Activation of CD30, a member of the TNFR, also stimulates the constitutive expression of the NF-κB superfamily. The mechanism of this expression is not clear, but in some cases, the gain of copies of REL due to aberrations that cause chromosome 2 (2p) gains [84] and the high-level expression of BCL3, a coactivator of NF-κB that binds to p50 in the nucleus in HRS cells, may be involved [85]. However, another study has suggested that the amplification of the 2p region is not always related to elevated expression of REL in diffuse large B-cell lymphoma (DLBCL) [86]. 

Epstein–Barr virus (EBV) is associated with several lymphoid malignancies. For example, EBV infection is related to Hodgkin’s lymphoma because EBV-encoded latent membrane protein 1 (LMP1), an integral membrane protein, activates both canonical and non-canonical signaling of NF-κB by inducing p100 processing to p52, similar to CD40, BAFFR, and Lymphotoxin receptor (LTR) [87,88,89,90]. LMP1 binds to tumor necrosis factor (TNF) receptor-associated factors (TRAFs) to activate NF-κB [89]. Mutations in A20, a negative regulator of NF-κB, were found in several lymphomas, including Hodgkin lymphoma, in a gene-wild analysis experiment [91]. A20 reduces HRS cell survival, functioning as a possible tumor suppressor [92].

Diffuse large B-cell lymphoma (DLBCL), which is the most common type of lymphoma in adults, is classified as activated B cell-like (ABC) DLBCL (marked by a poor prognosis) and terminal center B cell-like (GCB) types [93]. High levels of NF-κB have been found in ABC-DLBCL cells, but not in GCB DLBCL cells. This phenotype is associated with high constitutive IKK expression and IκB degradation. In contrast, the inhibition of NF-κB causes cell death and growth arrest in ABC-DLBCL cells [94]. Interestingly, an RNA interference screening study revealed that caspase recruitment domain family member 11 (CARD11) is an upstream regulator that is responsible for the IκB kinase activity. Along with BCL10 and MALT1, CARD11 leads to IKK activation and NF-κB translocation [95]. Mutations in genes that cause deregulation of NF-κB are found in ABC-DLBCL cells, and the main affected gene is A20, a downregulator of NF-κB. In contrast, missense mutations in *TRAF2* and *CARD11* express proteins that are able to activate NF-κB [96].

## 4. Bacteria, NF-κB and Gastric Cancer

Gastric adenocarcinoma is the second leading cause of cancer-related mortality worldwide [61]. Infection with *Helicobacter pylori* is the strongest recognized risk factor for this tumor, which is related to more than half of the cases [97,98]. *H. pylori* is an extracellular gram-negative bacterium that is considered an agent of gastritis and peptic ulcer [99,100]. A role for NF-κB in *H. pylori* infection has been suggested. *H. pylori* elicits the production of IL-8 in the gastric cell line MKN45 when the bacteria are in contact with the culture. NF-κB is the main activator of IL-8, which is the promoter of infiltrating neutrophils in the gastric mucosa and provokes chronic gastritis [101,102]. Keates et al. [103] also showed that the activation of NF-κB is related to the expression of IL-8. In the presence of the bacterial protein cytotoxin-associated gene A (CagA), the levels of IL-8 were increased when compared to non-cytotoxic bacteria [104]. CagA is a surface protein that is strongly associated with disease, since 75% of patients with gastroduodenal diseases and 100% of patients with duodenal ulcer harbor bacteria with this protein [105]. It was later found that cagA is part of a pathogenicity island (PAI) of approximately 40 kb inserted in the *H. pylori* chromosome (known as cagPAI) that encodes a type IV secretion system (T4SS) responsible for the secretion of proteins into the host cell that are also essential for the induction of IL-8 [106,107,108]. CagA is injected by T4SS into epithelial cells, where it is phosphorylated, triggering intracellular signaling responses [109]. The role of CagA as an inducer of IL-8 and NF-κB is controversial. Genes other than cagA present in the T4SS may be responsible for the induction of IL-8 [106,107]. However, Brandt et al. [110] confirmed that CagA induces NF-κB and IL-8 through the Ras-Raf-Mek-Erk axis in a time- and strain-dependent manner, but it does not cause differences in the cagA sequence between strains that could characterize this profile. CagA interacts and stimulates the ubiquitination of TAK1 in a TRAF6-dependent manner, which activates IKK, and, consequently, NF-κB [111]. The mechanism of activation of NF-κB by *H. pylori* is independent of lipopolysaccharide, but it requires at least six genes that are present in cagPAI but not cagA [112]. Bacteria can be recognized by the immune system via PRRs. Ectopic expression of TLR2 and 5 in HEK-293 cells incubated with Lipopolysaccharide (LPS) from *H. pylori* activated the expression of NF-κB, but the same result was not achieved with TLR4, in contrast to *Escherichia coli* LPS [30]. Conversely, the internal receptor Nod1 (CARD4) recognizes the specific gram-negative peptidoglycan secreted by T4SS and activates NF-κB [113,114]. These results indicate that NF-κB is not essentially activated by LPS of *H. pylori*, but by another molecular component in gastric cancer.

Fas-associated factor 1 (FAF1) is a pro-apoptotic protein, and its downregulation in gastric carcinoma indicates a poor prognosis. *H. pylori*, through NF-κB induction on tumor cells, reduces the expression of FAF1, promoting cell survival, and induces the inflammatory cytokines TNF-α and IL-8, which is associated to tumor growth [115]. IL-1β, another proinflammatory cytokine, is also stimulated by *H. pylori* infection. In mice, null mutants for IL-1β showed a 40% reduction in the expression level of p65, confirming the role of NF-κB in the inflammatory response in gastric cancer [116]. Both, IL-1β and *H. pylori* infection have been associated to gastric cancer carcinogenesis [117]. Thus, hematological and gastric cancers have a strong relationship with NF-κB through microorganisms and persistent infection. However, other types of neoplasia seem to support this signaling in an autocrine manner.

## 5. NF-κB and Breast Cancer

NF-κB plays an important role in mammary biology in all stages of development. Under normal physiological conditions, NF-κB is required for ductal development and regulation of mammary epithelial branching and proliferation [118]. Mammary stem cell expansion during pregnancy is induced though the activation of the receptor activator of NF-κB (RANK) pathway that positively regulates loboalveolar development [119]. Progesterone receptor (PR) upregulates the expression of RANK ligand (RANKL) in mammary epithelial cells (MECs), which release the ligand that binds to RANK expressed in other MECs, activating the RANK/NF-κB/cyclin D1 axis [120]. RANK or RANKL-knockout (KO) mice exhibit defective loboalveolar development and milk secretion during pregnancy [119] due to the downregulation of NF-κB activity, which is the main activator of the mitosis-inducing factor cyclin D1. NF-κB levels decrease during lactation, and its levels becomes almost undetectable during involution, which is marked by extensive apoptosis to achieve proper tissue remodeling [118,121].

Regarding breast cancer, constitutive activation of NF-κB contributes to cellular proliferation, angiogenesis, evasion of apoptosis, and is mostly described in Her2/neu. Liu et al. [122] reported that NF-κB is required for the initiation of Her2-positive murine mammary tumor growth. This transcription factor governs the initiation of Her2 tumors, and its inhibition was sufficient for decreasing the CD44-positive cell population and reduced the tumor microvessel density in models. When the Her2 murine cells expressed IκB mutant (S32A/S36A) that constitutively repress the NF-κB pathway, a reduction in mammosphere numbers and downregulation of the embryonic stem cell factors Sox2 and Nanog [122]. Merkhofer et al. [123] described the use of specific inhibitors and demonstrated that Her2 signal transduction leading to NF-κB activation occurred via the IKK complex in a PI3K/AKT-independent manner. Interestingly, IKKα played a more significant role than IKKβ, which is the most critical catalytic subunit. 

NF-κB showed significant support of cancer development and maintenance. Its role overtakes chemokine regulation because it dictates inflammatory aggravation, tumor microenvironment formation, and chemo- and radiotherapy resistance. Several reports have demonstrated that the activation of NF-κB signaling is a major marker of poor prognosis. Additionally, NF-κB is important for metastasis through epithelial to mesenchymal transition (EMT) [124,125,126,127,128,129,130].

During embryonic development, transcription factors that are related to EMT are responsible for controlling cell morphology and architecture of the neural crest and mesoderm formation. After embryogenesis, most of the genes that are involved in this mechanism are inactivated, but this entire regulatory pattern is recovered in cancer [131,132]. During this biological process, transcription factors, such as SNAIL, SLUG, ZEB1, SIP1, and TWIST1, repress adhesion molecules (E-cadherin, claudins, and occludins) and stimulate markers of the mesenchymal phenotype (N-cadherin, imentin, and ibronectin) [133,134]. During *Drosophila* sp. development, dorsal binds to DNA in a cooperative manner with dorsal switch protein1 (DSP1), and the two regulate twist promoter, which are responsible for the regulation of mesoderm differentiation [135]. Our group recently demonstrated that NF-κB transcriptionally regulates EMT-inducing factors [136]. Many studies have reported a link between increased NF-κB activity and a poor prognosis, in which the overexpression of NF-κB has been directly associated with an increase in metastasis [130,137,138]. In addition to the contribution of EMT to metastasis, NF-κB also induces the expression of UPA (urokinase-type plasminogen activator) and matrix metalloproteinases (MMP), which are effectors of extracellular matrix remodeling during cancer invasion [127]. For many years, efforts to treat cancer have focused on the destruction of tumor cells. The challenge now is to determine how and when some important physiological pathways must be specifically inhibited to attack the malignant cells. Hence, the NF-κB pathway is a promising target for cancer therapy.

## 6. NF-κB and Antitumor Immune Responses

T CD4+ lymphocytes are the main targets of many different immunotherapy trials. Some of these trials use monoclonal antibodies with specificity for inhibitory receptors expressed by T CD4+ cells. These specific antibodies lead to the inactivation of the inhibitory pathways, enabling the recovery of T CD4+ cell effectors functions, but also seem to reduce the number of Tregs [139], which are the major sub-type of T CD4+ controlling and driving immune response ablation, controlling other sub-types. Thus, NF-κB expression on T CD4+ cells might be important for the success or failure of these immunotherapies, as NF-κB supports the function of different TCD4+ subtypes. Other important mediators of the antitumor immune response also require NF-κB expression for its pro-inflammatory and effector functions, such as natural killer (NK) cells, innate-like lymphocytes (ILCs), Natural killer T (NKT) cells, and cytotoxic T CD8+ lymphocytes [44,140,141,142,143]. All of these studies substantiate that NF-κB is a transcription factor with important pro-inflammatory functions. 

The pro-inflammatory functions of NF-κB are well known, and it was surprising when studies using conditional knockouts (KO) for IKKβ and IKKγ (which will not have the NF-κB canonical pathway) in intestinal epithelial cells displayed excessive and abnormal immune responses in the gut [144,145]. In fact, recent studies have shown that NF-κB is crucial for Treg development and function [26,43,146], and the functionality of myeloid-derived suppressor cells [55]. Both of the cells are associated with anti-inflammatory regulatory immune responses and a poor cancer prognosis [147,148,149,150,151,152,153]. Moreover, NF-κB activation is also required for steady state dendritic cell migration to lymph nodes and endogenous antigen presentation, promoting Treg conversion from naive T CD4+ cells [154]. Tregs are crucial for inhibiting auto-immunity and inflammatory processes, but are also considered detrimental for anti-tumor immunity [155,156,157]. NF-κB also inhibits inflammasome caspase-1 activation, probably through the induction of anti-apoptotic proteins [158]. Moreover, the intensity of the NF-κB activating signal and its negative feedback, also seem to differ between acute and chronic inflammation [159] and might play a role in the genes that are induced in cells. Thus, a systematic understanding of how other pathways and transcriptional factors modify or contribute to the activity and outcome of NF-κB activation is crucial for better comprehending these discrepancies. Therefore, understanding how NF-κB plays a role in anti-tumor immunity can be very complex.

The role of NF-κB in cancer was systematically evaluated in myeloid cells (including dendritic cells, neutrophils, monocytes, mast cells, eosinophils, and macrophages) in different mouse models of tumorigenesis with contrasting results. Greten et al. showed that NF-κB ablation in myeloid cells (through IKKβ conditional deletion) in a colitis-associated tumorigenesis model was related to a significant reduction of colorectal tumor development and size [144]. The authors associated these findings with a decrease in different cytokines (IL-1β, IL-6, TNF-α, ICAM) in the milieu, accompanied by a lower proliferative profile of the cancerous cells. IL-1β, IL-6, and TNF-α are considered pro-inflammatory cytokines, and, likewise, NF-κB, can be both associated to tumor progression (promoting tumor growth, invasiveness, and metastasis) or rejection (through antitumor immunity and induction of cell death), depending on the context and the tumor model [160,161,162,163,164,165,166,167,168]. Hagemann et al. demonstrated that NF-κB expression in macrophages, both tumor-associated macrophages (TAM) and bone marrow-derived macrophages (BMDM), is an important factor for the polarization of these cells into a tumor supportive phenotype after in vitro co-culture with an ovarian cancer cell line [169]. NF-κB ablation increased STAT1 phosphorylation (activation) and polarization of the cells into a M1-like phenotype (inflammatory phenotype with tumoricidal activity and high expression of nitric oxide synthase (NOS)) [170,171]. Furthermore, after intraperitoneal injection of ovarian cancer cells and establishment of malignant ascites and tumors, the authors showed that infusion of macrophages with genetic ablation of IKKβ increased IL-12-dependent NK cell recruitment, leading to the inhibition of tumor growth. However, Hageman et al. [169] and Greten et al. [144] demonstrated a deleterious host effect that was associated with myeloid expression of NF-κB in different mouse tumor models, although the mechanisms were different. One was associated with reduced cytokine secretion at the tumor site [144], while the other was related to a phenotypic change in macrophages [169]. However, Yang et al. demonstrated that NF-κB ablation had contrasting effects on myeloid cells in a melanoma mouse model [172]. After the induction of the BRAF gene in melanocytes, the expression of NF-κB in myeloid cells was crucial for preventing tumor development in skin. NF-κB was also important for reducing the tumor burden in the lungs after the intravenous injection of syngeneic and allogenic melanoma, which was related to higher T CD8-mediated cytotoxicity (only in the allogeneic model) and DC maturation. Supporting the idea that NF-κB is important for the antitumoral immune response, Biswas et al. [173] and Saccani et al. [54] showed in a murine fibrosarcoma model that tumor associated macrophages (TAMs) had defective NF-κB signaling. Guiducci et al. [174] showed similar results. After toll like receptor 9 (TLR9) mediated NF-κB activation and IL-10 receptor blocking antibody, the infiltrating macrophages and DCs (which migrated toward an adenoviral expression of CCL16 on the tumors) would induce initial tumor necrosis. This initial response supported DC migration and tumor antigen presentation to T CD4^+^ cells, stimulating adaptive immunity and cytotoxic T cell response against the tumor. 

Regarding the immune response, many differences among these studies can explain the opposing results. The first important difference is that distinct tumor cells will not interact with their milieu and infiltrated immune cells in the same manner. Thus, NF-κB expression in the context of different signals afforded by ovarian cancer [169] or melanoma cells [172] can drive the expression of different genes. As a result, while NF-κB expression supported an M2-like phenotype in the co-culture model described by Hagemann et al. [169], this transcription factor appeared to have an opposite effect, at least in terms of tumoricidal activity, on the macrophages that were co-cultured with melanoma [172]. As previously discussed, NF-κB plays an important role in the development and function of immune cells, even when these immune cells have opposing functions, such as myeloid-derived suppressor cells [175] and M1-like macrophages [176]. Moreover, the site where the tumors were induced differed among all of the studies. For example, DCs and macrophages that were associated with the gut had important functional differences when compared with those that were found in other tissues, such as the dermis. The gut-associated macrophages and DCs were more prone to induce anti-inflammatory immune responses and had reduced or no ability to secrete pro-inflammatory cytokines [177,178,179,180,181]. Thus, the expression of NF-κB in myeloid cells from these two sites might be related to different polarizations of T CD4+ cells with different roles in the development of anti-tumor adaptive immunity. Unfortunately, T CD4+ cell polarization was not evaluated in these studies. In addition, the anti-inflammatory milieu of the mucosa from the gut may support the phenotype of myeloid-derived suppressor cells, but the types of myeloid-infiltrating cells in the tumors were not evaluated by Greten et al. [144]. Another important aspect that must be considered is the level of expression of NF-κB (and inflammatory cytokines) within the tumors. For example, different levels of TNF-α (which can be induced by NF-κB) is associated to cell death or proliferation during tumorigenesis, which outcome can also be affected by other cytokines and factors expressed in the milieu. Besides that, the regeneration rates of the organs can also affect the role of TNF-α in different cancer models. In this sense, TNF-α acts as an antitumorigenic cytokine in rapid regenerating tissues, like liver, while promotes tumorigenesis in slow regenerating tissues, like colon [72]. Thus, the stage of the disease, and its obvious transformed milieu, can also explain these discrepancies among the different studies. 

The role of NF-κB expression in T cell antitumor immunity was evaluated by Barnes et al. [182]. The authors used a transgenic and an immunogenic fibrosarcoma tumor model. The tumor cells that were injected subcutaneously in mice lacking NF-κB in T CD4+ cells (CD4 conditional deletion) were not rejected, as in the control mice. This phenomenon was related to a lower capacity of T cell secretion of TNF-α and Interferon-ϒ (IFN-γ), which are important cytokines mediating T cell antitumor immunity [183] and lower T cell-mediated tumor cytotoxicity. Another study [184] also highlighted that T cell recruitment is linked to a better prognosis in human lung cancer and rejection in a mouse model of immunogenic tumor. The authors showed that the constitutive NF-κB expression in lung cancer cells was related to chemokine secretion that will attract T cells. Through the generation of NF-κB gene expression signatures from tumor cell lines, the authors correlated the profile of NF-κB-induced chemokines with the presence of infiltrating T cells and a better prognosis in lung cancer. However, Guo et al. showed that NF-κB expression in pancreatic cancer cells was associated with IL-18-mediated proliferation and invasion of the tumor [161]. By blocking NF-κB by treatment with a pharmacological inhibitor (BAY11-7082) and injecting recombinant IL-18, a significant increase in mouse survival was observed. Based on results demonstrating the immunostimulatory role of IL-18, the authors concluded that the balance between the effects of IL-18-mediated antitumor immunity and its promotion of cancerous cell invasion could have favorable effects in the host by blocking NF-κB expression in cancer cells. Guo et al. showed that these results might be applicable to human patients, in which IL-18 found in plasma was related to good prognosis, while tumor site IL-18 was related to a poor prognosis [161]. These results highlight that the role of NF-κB in cancer cells can vary and require a better understanding prior to the design of a therapy. 

**Figure 3:**
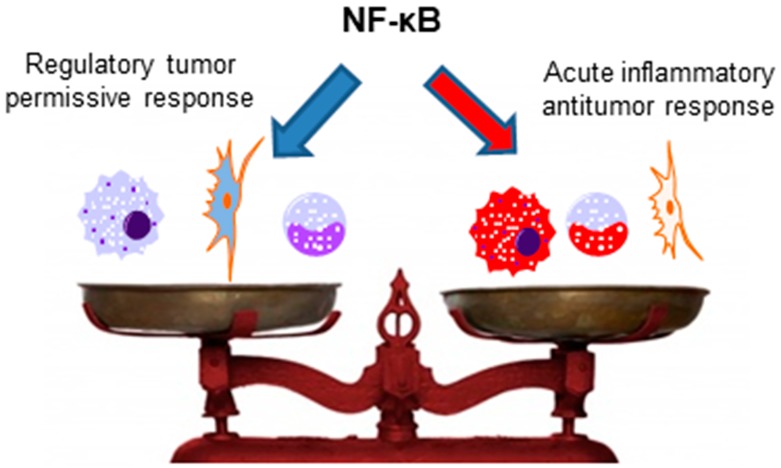
NF-κB role on tumoral immunity: NF-κB is important for the function of both pro-inflammatory and regulatory immune cells. As such, NF-kB can have a dichotomic role on tumoral immunity depending on the type of immune cells present in the cancerous tissue. If the majority of infiltrating cells are regulatory cells, like myeloid derived suppressor cells (MDSCs) or T reg, NF-kB expression will be associated to inhibition of antitumor immune response. However, NF-kB can play a crucial role on the anti-tumor immunity driven by infiltrating inflammatory cells, supporting perforin secretion by NK cells and macrophage ability to phagocyte and eliminate tumor cells.

The importance of NF-κB for antitumor immunity mediated by other immune cells, such as NK cells, NKT cells, γδ T cells, and T CD8+ cells, has not yet been analyzed, and studies with this aim would be interesting, as all of these cells have been previously described to present antitumor activities [142,185,186,187,188,189]. Interestingly, anergic T CD8+ cells, which are present in different cancers and are usually associated with impaired antitumor immunity [190], have ablated NF-κB activation [140]. Thus, the restoration of NF-κB activation in these cells could be a possible mechanism to circumvent the anergic phenotype and induce antitumor immunity. However, as previously discussed, NF-κB plays also an important role in Tregs and myeloid-derived suppressor cell functions; thus, the context, the type of infiltrating immune cells in the tumor, and the milieu are crucial for the role of NF-κB in the antitumor immunity (as simplified in Figure 3).

## 7. NF-κB and Perspectives for Therapy

The importance of understanding the intrinsic mediators (concomitant activation of other receptors, signaling pathways, and transcription factors) that drive the anti-inflammatory or pro-inflammatory roles of NF-κB in immune cells might be crucial to identify new targets for immunotherapy. Although the use of checkpoint inhibitors has shown promising results, they might not be sufficient because the anti-inflammatory stroma around cancer cells or even the absence of infiltrating T cells can compromise the success of this approach. Furthermore, the combination of different checkpoint inhibitors can have relevant side effects, and the sequence of administration might be crucial [191]. One promising technique is the combination of T CD4+ and myeloid immune cell-stimulating therapies that combat the regulatory and anti-inflammatory effects of infiltrating Tregs and TAMs simultaneously. NF-κB-activating receptors and other players may be good candidates for the activation of inflammatory myeloid cells or the inhibition of myeloid suppressor cells. Thus, the elucidation of the macrophages, monocytes, neutrophils, and DCs could represent an important supportive therapy for checkpoint inhibitors. 

Studies that are aiming to understand the role of NF-κB in antitumor responses have mainly focused on the canonical pathway, but targeting the non-canonical pathway may provide interesting perspectives. Recently, Yu et al. described the importance of the non-canonical NF-κB pathway for the function of myeloid-derived suppressor cells [55]. Another interesting study [192] has previously shown that IKKα, a regulator of the non-canonical NF-κB pathway, is also associated with the negative regulation of the NF-κB pathway by accelerating the turnover and removal of p65 and c-Rel from promoters of pro-inflammatory genes. Thus, the IKK complex may represent an interesting target to stimulate immune responses. In fact, each member of the NF-κB family may play specific roles in different models, and are, by themselves, promising targets [193,194,195,196]. 

The use of NF-κB inhibitors might be interesting, but its effects can be highly variable depending on the context and type of tumor. In relation to antitumor immunity, if the major cell type in the tumor microenvironment consists of regulatory cells, downregulation of NF-κB can increase antitumor immunity by inhibition of these regulatory cell functions. However, as NF-κB can also affect the effector function of other immune cells, it is possible that the overall response is not as effective as expected. Thus, specific factors that regulate NF-κB function in different immune cells can provide more appropriate targets. Finally, the combination of immunotherapy with radio and chemotherapy might also represent a promising way to circumvent the limitations that are associated with cancer treatment, as suggested by different studies [197,198,199]. In fact, the success of conventional therapy is sometimes related to the induction of antitumor immunity through the inflammatory and immune stimulatory effects of necrotic cancer cells (as a result of radio or chemotherapy treatment [200,201]), or through preventing the regulatory effects of specific immune cells, such as Tregs. Under conditions such as lymphopenia (generated by some chemotherapies), the ability of Treg cells to inhibit the effector functions of other T cells is reduced [202,203]. In addition, Duarte et al. [204] showed that under lymphopenic conditions, transferred Treg cells can be redirected to become effector cells, promoting autoimmunity (and probably also inducing antitumor immunity). 

Complex diseases, such as cancer, may require complex solutions, and more studies are needed to better understand antitumor immune responses. In this context, IL-10, which was categorized as a regulatory and anti-inflammatory cytokine, is now being described as an important mediator of antitumor immunity [205]. In the same way, different proinflammatory cytokines, like IL-6 and TNF-α, are described as tumor promoters and are related to poor prognosis [206]. A systematically evaluation of these discrepancies might generate a better understanding of the different types of cancers and open important paths to be followed. Another promising way to pursue is the development of combined therapies that can target diverse aspects to resolve multifactorial diseases, such as cancer.
